# Mesenchymal Stem Cells in Systemic Sclerosis: Allogenic or Autologous Approaches for Therapeutic Use?

**DOI:** 10.3389/fimmu.2018.02938

**Published:** 2018-12-14

**Authors:** Pauline Rozier, Alexandre Maria, Radjiv Goulabchand, Christian Jorgensen, Philippe Guilpain, Danièle Noël

**Affiliations:** ^1^IRMB, Univ Montpellier, INSERM, CHU Montpellier, Montpellier, France; ^2^Department of Internal Medicine, Multiorganic Diseases, Saint-Eloi Hospital, Montpellier, France; ^3^Clinical Immunology and Osteoarticular Diseases Therapeutic Unit, Lapeyronie University Hospital, Montpellier, France

**Keywords:** mesenchymal stem cell, systemic sclerosis, allogenic, cell therapy, bleomycin, HOCl

## Abstract

Systemic sclerosis (SSc) is a rare autoimmune disease, which is potentially lethal. The physiopathology of the disease is still incompletely elucidated although the role of fibroblasts, endothelial cells (ECs), immune cells. and the environment (i.e., oxidative stress) has been demonstrated. This is an intractable disease with an urgent need to provide better therapeutic options to patients. Mesenchymal stem cells (MSCs) represent a promising therapeutic approach thanks to the number of trophic and pleiotropic properties they exert. Among these, MSCs display anti-fibrotic, angiogenic, and immunomodulatory capacities that might be of interest in the treatment of SSc by acting on different processes that are dysregulated in the disease. In the recent years, the therapeutic effectiveness of MSCs has been demonstrated in different preclinical animal models and is being investigated in phase I clinical trials. Both allogenic and autologous transplantation of MSCs isolated from bone marrow or adipose tissue is being evaluated. The rationale for using allogenic MSCs in SSc, as well as in other autoimmune diseases, is based on the possibility that autologous MSCs might be altered in these diseases. In SSc, reports from the literature are controversial. Nevertheless, the role of the oxidative environment and of the crosstalk with neighboring cells (fibroblasts and ECs) on the functional properties of MSCs has been reported. Here, we review the preclinical and clinical data reporting the interest of MSC-based treatment in SSc and question the use of autologous or allogeneic MSCs in perspective of clinical applications.

## Introduction

Systemic sclerosis (SSc) is a rare autoimmune disease, which affects most frequently middle-age patients with a prevalence ranging from 100 to 300 per million depending on the country. The pathophysiology of SSc is still not completely understood even though three main axes of dysfunction are reported: fibrosis, vascular activation and immune abnormalities. The disease is characterized by vascular damage and diffuse fibrosis, which mainly affects skin and lung tissues but heart and digestive tract could also be involved (1). SSc is typically classified as limited or diffuse according to the extent and distribution of skin fibrosis (2). One of the earliest and most frequent symptom is the Raynaud's Phenomenon but vasculopathy is also responsible of other clinical signs including digital ulcers, pulmonary arterial hypertension, and telangiectasia (3). All of these symptoms are responsible for increased morbidity and lead to functional disability (reduced mouth opening and loss of hand function, for example), pain, and psychological consequences. This impacts not only the patient's quality of life but also reduces his life expectancy. In at least half of the cases, patients will die from SSc-related disorders and the other half from higher incidence of malignancies and cardiovascular diseases compared to the general population (4–6).

There is no curative treatment to date. Only symptomatic treatments are commonly proposed to patients to alleviate pain and improve function. Novel therapeutic strategies are being envisaged among which mesenchymal stem cells (MSCs)-based therapy, which is currently under evaluation in the clinics. The first clinical trial has evaluated the safety of MSCs-containing autologous stromal vascular fraction (SVF) (7) but the option of using allogeneic MSCs is also under evaluation since publications have reported potential alterations of MSCs from SSc patients. Here, we review the available literature on MSC-based cell therapies in SSc from pre-clinical models to clinical applications and discuss the interest of using allogeneic or autologous MSCs for future clinical trials.

## Generalities on Mesenchymal Stem Cells

MSCs are adult multipotent progenitor cells, which have been first identified in the bone marrow (BM) (8). In addition to bone marrow-derived MSCs (BM-MSCs), they have been described in different niches and isolated from several tissues, including adipose tissue (ASCs), umbilical cord (UC-MSCs), placenta, or dental pulp (9). Actually, MSCs have been proposed to be present in virtually all organs as pericytes, which show identical differentiation capacities *in vitro*. However, recent lineage-tracing experiments indicate that *in vivo*, pericytes do not contribute to other cell lineages (10). These results therefore challenge the concept that MSCs, or at least part of them, are pericytes behaving as multipotent tissue progenitors.

MSCs are defined by 3 criteria, as proposed by the International Society for Cell Therapy: (1) plastic adherence, (2) expression of the cell surface markers CD73, CD90, CD105, and lack of expression of the hematopoietic markers CD11b or CD14, CD19 or CD79α, CD34, CD45, HLA-DR, and (3) capacity of differentiation into adipocytes, chondrocytes, and osteoblasts (11). Along with their potential of multilineage differentiation, MSCs exert a number of paracrine functions: they support survival and differentiation of hematopoietic stem cells, induce cell proliferation and have anti-fibrotic, anti-apoptotic, pro-angiogenic, anti-bacterial, and anti-inflammatory effects (12). These effects are mediated primarily through the secretion of soluble mediators but can be enhanced upon contact of MSCs with the target cells (13). The soluble factors are released in the extracellular milieu or within extracellular vesicles that protect them from degradation and allow their transfer throughout the organism [for review, see (12, 14)]. Although, MSCs from different tissue sources share similar properties, they may display some differences in their differentiation potential or immunomodulatory capacity (15, 16).

Thanks to this pleiotropic activity, the therapeutic efficacy of MSCs has been investigated in different pathological conditions, from inflammatory diseases to acute or degenerative diseases. So many different applications as rheumatic diseases (17), stroke (18), lupus and scleroderma (19), heart diseases (20), or bone defects (21) have been evaluated. To date, thousands of patients have been enrolled in hundreds of clinical trials and safety of MSC injection has been proved. In 2016, a meta-analysis has reported no acute toxicity, death, infection, organ systemic failure or risk malignancy after MSC implantation (22). Only transient fever was frequently observed. Efficacy of MSC-based treatments as compared to standards of care is still however under evaluation but promising results have been achieved mostly in phase II trials. For SSc, very few trials have been initiated and results from a couple of registered phase I/II clinical trials are still pending.

## Therapeutic Effect of MSCs in Preclinical Models of SSc

Before the initiation of clinical trials, the interest of using MSCs in the treatment of SSc has been evaluated in different preclinical models. Three models have been used: the model of bleomycin-induced pulmonary fibrosis, the model of hypochlorous acid (HOCl)-induced SSc and the tight skin (Tsk1/+) mouse model of SSc.

### The Model of Bleomycin-Induced Fibrosis

The model of bleomycin-induced fibrosis is the most widely used model to replicate scleroderma, or dermal or pulmonary fibrosis in mice or rats. According to the mode of administration, fibrosis can be induced in skin when bleomycin is injected daily sub-dermally or in lung after a single intra-tracheal instillation. Fibrosis in lung, skin, and internal organs may be induced via the use of osmotic pumps that deliver a constant amount of bleomycin over a number of days (23, 24). It is believed that bleomycin causes breaks in DNA, resulting in overproduction of reactive oxygen species (ROS) and inflammatory response that activate fibroblasts and subsequently fibrosis formation. Importantly, the bleomycin models replicate some of the earliest patterns of SSc but do not present with the typical clinical signs and autoantibody patterns of SSc.

The therapeutic potential of MSCs has been first demonstrated in the mouse model of lung fibrosis. One intravenous injection of 500,000 allogenic BM-MSCs on the day of bleomycin instillation was shown to reduce collagen content and inflammation in the lungs (25). At day 14 after injection, the authors could detect engraftment of BM-MSCs in areas of bleomycin-induced injury where they adopted an epithelium-like phenotype. Another study confirmed that systemic injection of allogenic BM-MSCs after intra-tracheal instillation of bleomycin protected mice from injury and fibrosis through the suppression of inflammation (26). They also reported that BM-MSCs differentiated into distinct lung cell phenotypes and secreted chemokines that might have attracted endogenous cells. Due to the relatively low numbers of transplanted MSCs and persistence of some injury in BM-suppressed animals, the authors suggest that both endogenous and exogenous MSCs likely contribute to the repair process. Another study comparing minimally cultured (2 h) to conventionally cultured (9 days) BM-MSCs indicated that both types of cell preparations ameliorated as efficiently inflammatory and progressive fibrotic lung injury (27). Minimally cultured BM-MSCs had a higher proliferative capacity, expressed higher levels of stem cell markers, and chemokine receptors but lower levels of type I procollagen, α-smooth muscle actin (α-sma) and transforming growth factor-β (TGFβ) that might be more advantageous for cell-based therapy.

The therapeutic effect of BM-MSCs has been also confirmed in rat models of bleomycin-induced lung fibrosis. One intravenous injection of five millions of BM-MSCs resulted in decreased levels of TGFβ1, platelet-derived growth factor-A (PDGF-A), PDGF-B, insulin growth factor-1 (IGF1), and in lower collagen content in lungs (28). Another report has shown that systemic injection of syngeneic BM-MSCs ameliorated fibrosis and decreased inflammatory and angiogenic markers as well as nitric oxide metabolites (29).

In a dermal fibrosis model induced by daily subcutaneous injection of bleomycin for 4 weeks, syngeneic BM-MSCs were injected subcutaneously into the lesion skin every day (30). Treatment with BM-MSCs resulted in a basket-weave organization of collagen arrangement similar to normal skin, with few inflammatory cells and α-sma-positive myofibroblasts as well as down-regulation of TGFβ, type I collagen, and heat-shock protein 47 (HSP47) expression in skin.

The impact of MSCs from different species or tissues on treatment efficacy has been evaluated in the mouse model of bleomycin-induced lung fibrosis. Human UC-MSCs injected systemically 24 h after bleomycin instillation in SCID mice were reported to reduce inflammation markers, collagen content, and inhibit expression of TGFβ, interferon-γ (IFNγ), tumor necrosis factor-α (TNFα) (31). In this study, improvement of tissue remodeling was shown with increased levels of matrix metalloproteinase-2 (MMP-2) and decreased levels of their endogenous inhibitors, tissue inhibitor of MMP (TIMP). Of interest, another report demonstrated that both human amnion- and chorion-derived fetal MSCs displayed similar reduction in the severity of bleomycin-induced lung fibrosis as allogeneic murine amnion- and chorion-derived fetal MSCs (32). Amniotic fluid MSCs were shown to inhibit collagen deposition and to preserve pulmonary function, which could be related to transient increase of MMP-2 (33). Cells were observed to localize within fibrotic lesions with a preferential targeting to the area of fibrosis. Similarly, compared to BM-MSCs, human amnion-derived MSCs reduced collagen deposition and increased MMP-9 activity (34). Finally, ASCs attenuated lung fibrosis induced by repetitive intra-tracheal administrations of bleomycin, namely hyperplasia of Club cells (Clara cells) and cuboidal alveolar epithelial cells, infiltration of the perialveolar ducts by inflammatory cells, septal thickening, enlarged alveoli, and extensive fibrosis (35). It also led to suppression of epithelial cell apoptosis and expression of TGFβ suggesting that irrespective of the tissue or species origin, MSCs are potent inhibitors of lung fibrosis.

The time of cell injection after disease induction may be one important factor to control for better efficacy of cell therapy. Most frequently, MSCs have been implanted at the time or within the few hours after bleomycin instillation. Interestingly, early treatment (day 0) with murine or rat BM-MSCs on the day of bleomycin instillation resulted in a significant reduction of fibrotic changes that was not seen when BM-MSCs-based treatment was delayed (day 7) (25, 36). Similarly, two injections of low (2.8 × 10^6^cells/kg) or high (5.6 × 10^6^cells/kg) doses of human BM-MSCs at day 7 and 15 after fibrosis induction did not lead to improvement of lung function or rescue of damage tissue (37). Injection of autologous ASCs via the trachea at day 15 of the disease did not improve the severity of lung injury but prevented further aggravation of lung damage (38). In this report, the majority of ASCs did not penetrate inside the lung region at week 3 but some cells had sprouted deep into the distorted architecture of the lung at week 6 after disease induction. By contrast, injections of murine amniotic fluid-derived MSCs at either day 0 or day 14 were both efficient to inhibit fibrosis indicating improvement of the disease in both acute and chronic remodeling events (33). Injections of amniotic MSCs or BM-MSCs at day 3 or 6 were even more efficient to reduce lung inflammation than the treatment at day 1 (39). In addition, in a model of repeated injections of bleomycin at day 0 and 7, BM-MSCs injected at day 10 were beneficial in terms of collagen deposition reduction and MMP-9 down-regulation (34).

The age of the donors may be another parameter that might change the therapeutic potential of MSCs. One study compared ASCs from aged (>22 months) and young (4 months) mice (40). At day 21 after bleomycin instillation, mice receiving young ASCs exhibited decreased fibrosis, MMP-2 activity, oxidative stress, and markers of apoptosis vs. untreated controls. Improved treatment with young-donor ASCs was associated with decreased mRNA expression of MMP-2, IGF receptor, and protein kinase B (AKT) activation.

For more a decade since 2003, the skin or lung bleomycin-induced model of local fibrosis was the single model used to evaluate the therapeutic effect of MSCs. However, it does not reproduce the main characteristics of the diffuse human SSc. With respect to the systemic nature of the disease, relevant models of systemic fibrosis have to be used to better evaluate the efficacy of MSCs in models closer to human SSc.

### The Model of Bleomycin-Induced Lung and Skin Fibrosis in Aged Mice

A study relied on the use of an established aged (18–22-month-old) mouse model of bleomycin-induced lung fibrosis to test the hypothesis that fibrosis may develop simultaneously in multiple organs and notably affects lung, skin and wound healing. Mice developed irreversible lung and skin fibrosis as well as delayed wound closure at day 21 (41). In this model, intravenous single injection of allogeneic ASCs attenuated lung fibrosis as evaluated by semi-quantitative Ashcroft score on Masson's-trichrome stained histological sections. ASCs also accelerated wound healing as shown by increased total wound size and wound gaps. This effect was associated with higher levels of caveolin-1 and lower level of α_v_integrin, TNFα, and miR-199-3p in lung and skin wounds. These results therefore support the hypothesis that ASCs may prevent systemic fibrosis and enhance wound healing but rely on the use of aged mice that are expensive and difficult to get.

### The Murine Model of HOCl-Induced SSc

In order to evaluate the therapeutic effect of MSCs in a systemic model of scleroderma, the murine model of HOCl-induced SSc is relevant, cheap and reproducible. After daily intradermal injections of HOCl for 6 weeks, mice develop a systemic disease with the main features of the human form, including skin and lung fibrosis, vascular abnormalities and the production of Anti-Scl-70 autoantibodies, which are anti-nuclear topoisomerase antibodies mainly detected in diffuse systemic scleroderma (42). Our group demonstrated the therapeutic effect of a single intravenous injection of syngeneic BM-MSCs in preventive (day 0) but also curative (day 21) approaches (43). Interestingly, the dose escalation study revealed that the best improvement of both skin and lung fibrosis was obtained with the lowest dose of 2.5 × 10^5^ BM-MSCs. We also compared different sources of cells, i.e., allogenic, xenogeneic, and syngeneic BM-MSCs as well as xenogeneic ASCs, which seemed to be the most potent as compared to BM-MSCs (44). The therapeutic effect was observed on all features of SSc, including reduction of fibrotic, inflammatory. and oxidative markers, but also reduction of autoantibodies Scl-70 while matrix remodeling markers were increased. Finally, infused GFP-expressing allogeneic BM-MSCs were entrapped in the lungs where they were detected at days 1 and 2 but not at day 7. Interestingly, either murine allogenic or human xenogeneic BM-MSCs did not migrate to the skin although skin fibrosis was reduced, indicating a systemic effect of BM-MSCs (44, 45).

### The Model of Tight Skin (Tsk1/+) Mouse

The tight skin 1 (Tsk1/+) mouse occurred as a spontaneous mutation in fibrillin-1, which controls the biologically active levels of TGFβ through binding to the latent TGFβ binding proteins (LTBP) 1 and 4 (46). It has therefore been suggested that activation of the TGFβ signaling axis is involved in the development of the phenotype. This genetic model of SSc is characterized by hyperplasia of the sub-cutaneous loose connective tissue with abnormal dermis, osteopenia and deregulation of the interleukin-4 (IL4)/IL4 receptor (IL4R) signaling pathway. Interestingly in this model, MSCs have been shown to ameliorate osteopenia by rescuing impaired lineage differentiation of the recipient BM-MSCs (47). In this interesting study, the authors further demonstrated that MSC-derived extracellular vesicles containing miR-151-5p rescued the disease phenotypes via regulation of the IL4R pathway in recipient BM-MSCs.

Altogether, the beneficial effect of MSCs-based treatment has been proved in the different models of local or systemic scleroderma (Table 1). Some clues of their mechanism of action have also been investigated as discussed below.

**Table 1:** Summary of studies on the role of mesenchymal stem cells in preclinical models of systemic sclerosis.

**Model/species**	**Number and origin of MSCs**	**Route of injection**	**Injection time**	**Main results**	**Mechanisms**	**References**
Bleomycin intratracheal/mouse	5 × 10^5^ allogenic BM-MSCs	IV	d0 or d7	Reduction of collagen content and inflammation in lungs after treatment at d0	ND	(25)
Bleomycin intratracheal/mouse	5 × 10^5^ syngeneic BM-MSCs	IV	H6	Reduction of inflammation and fibrosis in lungs	Differentiation into distinct lung cell phenotypes	(26)
Bleomycin intratracheal/mouse	5 × 10^5^ syngeneic BM-MSCs	IV	d3	Reduction of collagen content and inflammation in lungs	ND	(27)
Bleomycin intratracheal/rat	5 × 10^6^ syngeneic BM-MSCs	IV	H12	Reduction of TGFβ1, PDGF-A, PDGF-B, IGF1 and collagen content in lungs	MSC differentiation into alveolar epithelial cells	(28)
Bleomycin intratracheal/rat	1 × 10^6^ syngeneic BM-MSCs	IV	d4	Diminution of inflammation, collagen content, angiogenic markers and nitric oxide metabolites in lungs	ND	(29)
Bleomycin subcutaneous/mouse	1 × 10^6^ syngeneic BM-MSCs	SC	Daily injection during 4 weeks	Improvement remodeling matrix responsible for normal collagen arrangement in skin. Reduction of inflammation and α-sma-positive myofibroblasts	Down-regulation of TGFβ, type I collagen and HSP47 expression	(30)
Bleomycin intranasal/mice	1 × 10^6^ human UC-MSCs	IV	d1	Reduction of inflammation, collagen content and TGFβ expression and improvement remodeling of matrix	ND	(31)
Bleomycin intratracheal/mouse	4 × 10^6^ IP or 1 × 10^6^ IV ou IT xenogeneic & allogeneic amnion- and chorion-derived fetal MSCs	IP or IV or IT	d0	Reduction of lung fibrosis	ND	(32)
Bleomycin intratracheal/mouse	1 × 10^6^ murine amniotic fluid MSCs	IV	d0 or d14	Inhibition of collagen deposition and preservation of pulmonary function	ND	(33)
Bleomycin intranasal/mouse	Human BM-MSCs or amnion-derived MSCs	IV	d0 and d7	Reduction of inflammation and collagen content in lungs	ND	(34)
Bleomycin intratracheal/mouse (every 2 weeks, 8 doses in total)	5 × 10^5^ human ASCs	IP	4 doses at time of bleomycin injection	Reduction of lung fibrosis and inflammation	ND	(35)
Bleomycin intravenous/rat	5 × 10^5^ syngeneic BM-MSCs	IV	d1 or d7	Reduction of lung fibrosis only after treatment at d1	ND	(36)
Bleomycin intratracheal/rat	2.8 × 10^6^ or 5.6 × 10^6^ human BM-MSCs /kg	IV	d8 or d15	Safety of MSC injection No amelioration of disease	ND	(37)
Bleomycin intratracheal/rat	Autologous ASCs	IT	d15	No improvement but prevention of lung damage aggravation	ND	(38)
Bleomycin intratracheal/mouse	5 × 10^5^ syngeneic BM-MSCs	IV	d1 or d3 or d6	Reduction of lung inflammation & fibrosis after d3 or d6 treatment	ND	(39)
Bleomycin intratracheal/mouse	5 × 10^5^ syngeneic ASCs from old or young mice	IV	d1	Only young ASCs induced lower lung fibrosis, oxidative stress and apoptosis	Lower levels of MMP-2, IGFR and AKT activation	(40)
Bleomycin intratracheal/mouse	5 × 10^5^ syngeneic ASCs	IV	d1	Reduction of lung & skin fibrosis Acceleration of wound healing	Decreased miR-199-3p and increased caveolin-1 in lungs and skin	(41)
HOCl intradermic injection/mouse (daily, 42 days)	2.5 × 10^5^ syngeneic or allogeneic BM-MSCs, or human ASCs & BM-MSCs	IV	d0 or d21	Reduction of fibrotic, inflammatory and oxidative markers in skin & lungs. Improvement of matrix remodeling.	ND	(43) (44) (45)
Tsk1/+ mouse	1 × 10^5^ allogenic BM-MSC /kg bodyweight	IV	8 weeks	Improvement of osteopenia	Downregulation of the IL4R pathway by miR-151-5p in MSC-EV	(47)
Bleomycin intratracheal/rat	2.5 × 10^6^syngeneic BM-MSCs	IV	d0 or d7	Reduction of alveolitis, pulmonary fibrosis and oxidative stress	Conversion of BM-MSC into type II alveolar epithelial cells	(48)
Bleomycin intratracheal/rat	0.2mL allogenic BM-MSC supernatants	IT	H6 and d3	Reduction of collagen content, inflammation and fibrosis in lungs	ND	(49)
Bleomycin intratracheal/mouse	5 × 10^5^ allogenic BM-MSCs	IV	d0	Reduction of IL1α lung level	IL1RN expressing MSCs antagonizing IL1α	(50)
Bleomycin intranasal/mice	5 × 10^4^ allogenic or HGF KO BM-MSCs/g bodyweight	IV	H6 or d9	Reduction of lung fibrosis and inflammation, and increase of HGF	HGF release	(51)
Bleomycin intratracheal/mouse	2.5 × 10^5^ syngeneic OSM- preconditioned BM-MSCs	IT	d3	Diminution of inflammation and fibrosis in lungs and improvement of respiratory function	Production of high level of HGF	(52)
Bleomycin intratracheal/mouse	2.5 × 10^5^ syngeneic hypoxia-preconditioned BM-MSCs	IT	d3	Improvement of lung function and matrix remodeling. Decreased pro-inflammatory and fibrotic factors in lungs	Anti-apoptotic	(53)
Bleomycin intratracheal/mouse	2 × 10^5^ NAC-pretreated human embryonic MSCs	IV	d1	Decrease of inflammation and lung fibrosis.	Increased antioxydative capacity of MSCs	(54)
Bleomycin intratracheal/mouse	5 × 10^5^ human BM-MSCs overexpressing let7d	IV	d7	Reduction of collagen content and inflammation in lungs	Let7d over-expression	(55)
Bleomycin intratracheal/mouse	1 × 10^6^ xenogenic UC-MSCs over-expressing ACE2/kg bodyweight	IV	d3	Decrease of collagen content, fibrotic and pro-inflammatory factors and increase of anti-oxidative mediators	ACE2 over-expression	(56)
Bleomycin subcutaneous/mice (daily, 21 days)	1 × 10^6^ Trx-1-overexpressing BM-MSCs	SC	Daily	Reduction of skin fibrosis and apoptosis, promotion of BM-MSC survival and differentiation into endothelial cells	TRX1-mediated inhibition of oxidative stress	(57)

*ACE2, angiotensin converting enzyme 2; AKT, protein kinase B; ASCs, adipose tissue-derived mesenchymal stem cells; BM-MSCs, bone marrow mesenchymal stem cells; HGF, hepatocyte growth factor; HOCl, hypochlorite; IGF, insulin growth factor; IGFR, IGF receptor; IL, interleukin; IL1RN, interleukin 1 receptor antagonist; IP, intraperitoneal; IT, intratracheal; IV, intraveinous; KO, knock-down; MMP, metalloproteinase; MSC-EV, mesenchymal stem cell-derived extracellular vesicles; NAC, N-acetylcystein SC, subcutaneous; OSM, onconstatin M; PDGF, platelet-derived growth factor; TGFβ, transforming growth factor; Trx-1, thioredoxin 1; Tsk1, tight skin; UC-MScs, umbilical cord-derived mesenchymal stem cells*.

## Mechanisms of Action of MSC-Based Therapy in SSc

The capacity of MSCs to differentiate into several cell types of musculoskeletal tissues was the first property that has attracted the attention of researchers and clinicians (58). Since then, several mechanisms of action have been proposed to play important roles in various disorders and diseases via the secretion of many soluble mediators as summarized in (12). Besides anti-apoptotic and anti-bacterial functions, the support of hematopoietic stem cells, the chemoattracting effect, the proliferative, and protective role of MSCs has been largely exemplified. In SSc, the main functions of MSCs are proposed to be anti-inflammatory to counteract the dysregulation of the immune system, anti-fibrotic to down-regulate the excessive production of collagen associated to thickening of skin and internal organs but also pro-angiogenic to counteract the widespread vasculopathy. A number of reviews have discussed the interest of using MSCs for the treatment of SSc (19, 59, 60). In the next paragraph, we focused our attention on the mechanisms that have been deciphered specifically in preclinical models of SSc.

Systemic administration of MSCs results in their homing in lungs and cell engraftment is increased in lungs from bleomycin-induced mice, in particular in areas of injury where they adopt an epithelial cell morphology and display anti-oxidative role (25, 48). Of interest, in the HOCl-induced systemic model of SSc, both human or green fluorescent protein (GFP)-positive murine BM-MSCs were shown to be retained in the lungs of mice after intravenous administration while no cells were detected in the skin suggesting a systemic effect of infused BM-MSCs (43, 45). In addition, tracheal instillation of BM-MSC supernatants decreased the number of apoptotic cells, collagen content, inflammation and fibrotic scores in lungs in the model of bleomycin-induced lung fibrosis (49). These results suggest that BM-MSCs preferentially act through the secretion of soluble mediators that are distributed throughout the body but they can also act directly in contact with damaged lungs where they preferentially reside.

**Figure 1:**
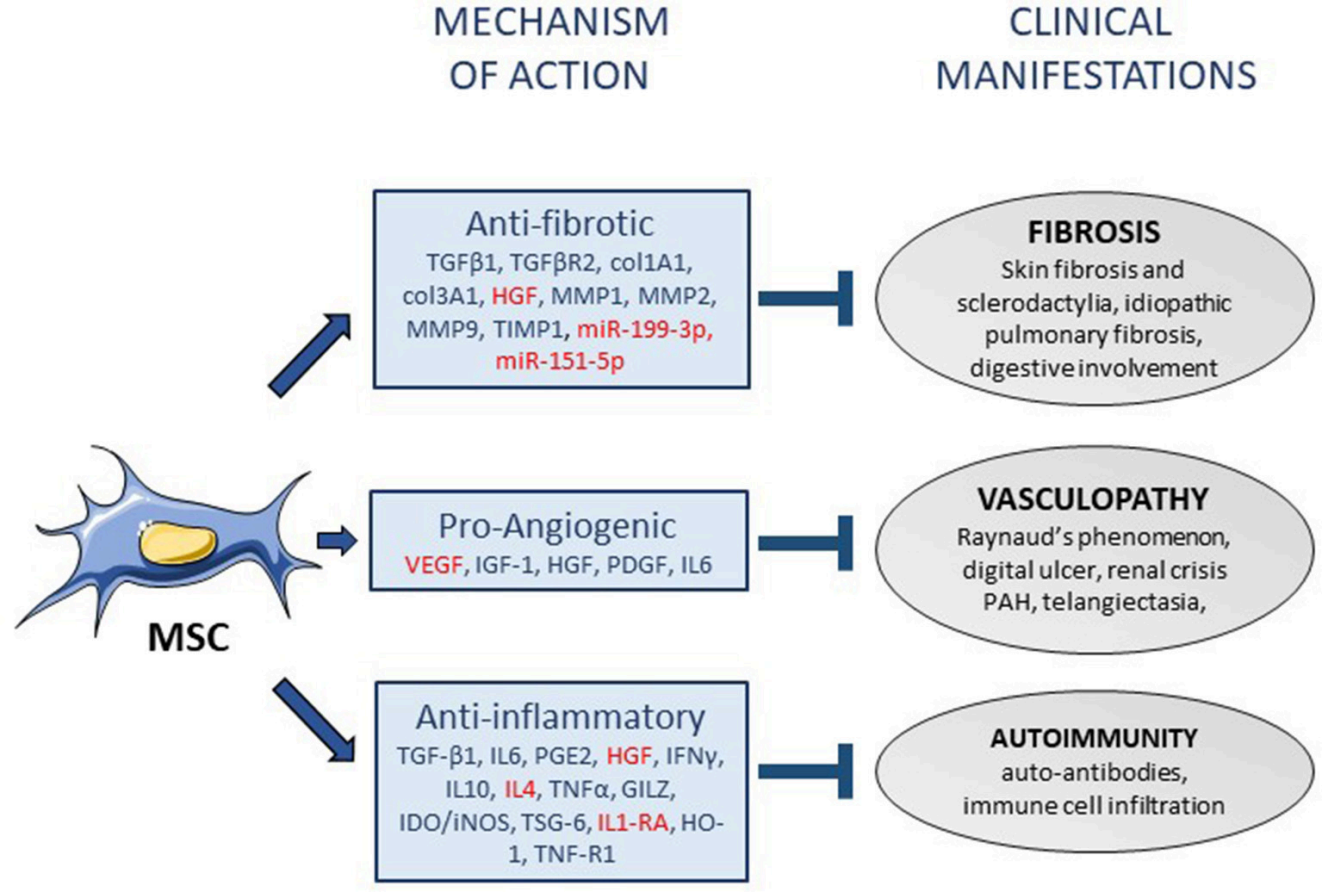
Mechanisms of action of mesenchymal stem cells in systemic sclerosis. Mesenchymal stem cells (MSC) display various functions that might participate to their mechanisms of action in the treatment of systemic sclerosis (SSc), through the release of soluble mediators (arrows). The mediators that have been associated with anti-fibrotic, pro-angiogenic or anti-inflammatory functions of MSCs are listed in the squares (in red: mediators specifically identified in SSc). Three mechanisms of action of MSCs can be associated with the improvement of the three main clinical manifestations of SSc (blind-ended arrows). PAH, pulmonary arterial hypertension.

Some secreted mediators involved in their therapeutic effect including IL1-RA, hepatocyte growth factor (HGF) have been identified in murine models of lung fibrosis (50, 51). However, novel strategies of MSC-based treatments have been tested in order to improve their efficacy by enhancing survival after *in vivo* administration or increasing their trophic action. Indeed, preconditioning of BM-MSCs before administration was shown to enhance their therapeutic efficacy. Namely, oncostatin M- or hypoxia-preconditioned BM-MSCs improved pulmonary respiratory function thanks to decreased inflammatory and fibrotic markers as compared to unconditioned BM-MSCs and this effect was mediated at least through the upregulation of HGF (52, 53). Pre-treatment of BM-MSCs with N-acetylcysteine was able to increase both treatment efficacy in a murine model of bleomycin-induced lung injury and BM-MSC survival through the production of anti-oxidant factors (54). In order to improve their therapeutic function, strategies to over-express factors have also been tested. Overexpression of let7d, which is a microRNA known to have anti-fibrotic effects, in BM-MSCs resulted in slight decrease of collagen content in lungs and improved mouse survival when injected at day 7 in bleomycin-induced mice while naïve BM-MSCs did not have any beneficial effect (55). Similarly, human UC-MSCs over-expressing angiotensin-converting enzyme 2 (ACE2) were more efficient than naïve UC-MSCs to decrease collagen content, fibrotic, and pro-inflammatory factors while increasing anti-oxidative and anti-inflammatory mediators (56). Thioredoxin 1 (Trx-1)-overexpressing BM-MSCs were also shown to inhibit apoptosis and fibrosis under hypoxic conditions and to promote the formation of tubular-like structures by endothelial cells in bleomycin-induced lung injury (57). Indeed, strategies for enhancing MSCs survival and/or efficiency have been demonstrated to be of interest in preclinical models of SSc. A summary of the potential (or proven) mechanisms of action of MSCs in the treatment of SSc is provided in Figure 1.

## Characterization of MSCs from SSc Patients

Early studies evaluating the phenotypic and functional characteristics of MSCs from SSc patients reported no major difference as compared with MSCs from healthy donors. Indeed, BM-MSCs from two patients with SSc have been compared to those of healthy donors 10 years ago (61). No major differences in their proliferative rate, adipogenic or osteogenic differentiation potential, expression of surface markers and immunosuppressive properties were reported between the two sources of BM-MSCs. Some years later, similar data were obtained using BM-MSCs from 12 scleroderma patients in comparison with 13 healthy controls (62). SSc and healthy BM-MSCs displayed the same phenotype, identical capacities to proliferate and form CFU-F, similar capacities to differentiate into adipogenic and osteogenic lineages, equal capacities to support long-term hematopoiesis, and similar anti-proliferative properties. SSc ASCs were also shown to have comparable phenotypic markers and functional characteristics (proliferative and differentiation potential) as healthy donor ASCs (63). However, this cohort of six SSc patients was characterized by cutaneous local forms of scleroderma (generalized morphea, linear and/or plaque scleroderma) and not diffuse systemic sclerosis. More recently, no difference in proliferative capacities and potential to support endothelial cell tube formation was noticed between ASCs from ten patients with diffuse SSc or eight healthy donors (64).

By contrast, other studies have reported some differences. As an example, MSCs isolated from the skin of scleroderma patients expressed more PDGF-R than those from healthy donors, resulting in the production of higher levels of ROS (65). Nevertheless, SSc-MSCs were still able to improve the antioxidant defenses by counterbalancing ROS accumulation. More recently, the impact of the pro-oxidant environment exerted by SSc sera on BM-MSCs has been investigated. Exposure of BM-MSCs to SSc sera enhanced their antioxidant capacities as well as their osteogenic and adipogenic potentials while their immunosuppressive functions were reduced (66). With the objective to evaluate the consequence of TGFβ activation in SSc, analysis of several members of the TGFβ family has been undertaken in BM-MSCs from nine patients with diffuse SSc (67). A highly significant increase in mRNA and protein levels of TGFβ receptor II (TβRII) was detected in SSc BM-MSCs as compared to healthy controls. Moreover, in response to TGFβ activation, the production of type I collagen and Smad-3 phosphorylation was up-regulated in SSc BM-MSCs and also in SSc fibroblasts. An earlier study reported that nerve growth factor receptor (NGFR)-positive BM-MSCs, a sub-population of MSCs with high proliferative and clonogenic potentials, were less numerous in BM from SSc patients than from healthy controls (68). They displayed lower clonogenic potential (10-fold less), had a reduced fold expansion rate (105-fold less), showed signs of rapid aging and stress and, never differentiated into adipocytes or osteoblasts. In line with these data, Cipriani and coauthors have described that BM-MSCs from SSc subjects expressed up-regulated α-sma and transgelin (TAGLN or SM22α) genes and displayed reduced proliferative activity and migration potential (69). An increase in senescence markers (senescence-associated β-galactosidase (SA-βGal), p21, IL6, TGFβ) was also observed while their immunosuppressive activity on lymphocyte proliferation and regulatory phenotype induction was retained (70). Interestingly, the same team showed an impaired crosstalk between endothelial cells (ECs) and BM-MSCs (71). In coculture conditions, SSc ECs induced increased levels of VEGF-A in SSc MSCs and of TGFβ, PDGF-R, α-sma, type I collagen in both SSc and healthy MSCs. Despite the production of high levels of VEFG-A by SSc MSCs, they had lower angiogenic performance. The authors also detected increased expression of TGFβ and PDGF-BB in SSc ECs when co-cultured either with MSCs from healthy or SSc individuals. This impaired crosstalk between ECs and MSCs suggested that ECs may be involved in the early steps leading to fibrosis by producing factors that induce a phenotype switch of MSCs toward myofibroblast cells. In another study, SSc BM-MSCs exhibited altered differentiation into contractile and synthetic vascular smooth muscle cells when stimulated by connective tissue growth factor (CTGF) or b-FGF. Moreover, stimulation with TGFβ1 induced a myofibroblast-like phenotype with high production of α-sma and collagens, higher migration capacity but less proliferative capacity (72). Accordingly, SSc BM-MSCs expressed up-regulated levels of proangiogenic factors including VEGF, stromal derived factor-1 (SDF-1) and C-X-C chemokine receptor (CXCR4) (73). However, they noticed that SSc BM-MSCs promoted angiogenesis and improved capillary morphogenesis as compared to healthy MSCs.

A unique study has identified altered functions in SSc ASCs (74). No alteration in their phenotype or differentiation potential was noticed but their proliferative rate, metabolic activity and migration capacity were reduced as compared to healthy matched control ASCs. However, no data on the expression of TβRII or other fibrotic markers as well as on their immunosuppressive and angiogenic functions were available. Further functional analysis is therefore required to fully decipher the therapeutic potential of ASCs isolated from SSc patients.

## Clinical Data on MSC-based Treatment in SSc

### Case Reports Using Fat Injection

In recent decades, fat tissue grafting has been used to treat skin atrophy or fibrosis thanks to its biocompatibility and property of filling but also to its high content in multipotent stem or progenitor cells with regenerative potential. The first report on the implantation of fat in hands of patients who presented Raynaud's phenomenon was published in 2014 (75). Among the thirteen treated patients, nine patients suffered from scleroderma. There were no complications and the treatment showed some evidence of perfusion. In 2015, another study reported a significant improvement of digital ulcerations, hand grasping, and pain after injection of adipose tissue-derived cells into fingers of 15 SSc patients suffering from digital ulcers (76). More recently, the effect of one local injection of autologous SVF associated with platelet-rich plasma into malar and perioral areas in six patients showed improvement of skin elasticity and vascularization (77). Some indication of efficacy was suggested by these case reports but the role of ASCs contained within the SVF could not be drawn.

### Case Reports Using MSCs

The first patient who received BM-MSCs in the treatment of progressive diffuse SSc has been reported in 2008 (78). A young female patient had severe disease, refractory to all immunosuppressive drugs. At time of implantation, she presented with six painful ulcerations and received 6 × 10^7^ intravenous administration of allogeneic haploidentical-related donor BM-MSCs. No adverse events were reported and 3 months after treatment, a significant decrease in the patient's painful ulcerations was measured. Vascular improvement in the blood circulation of hands and fingers was noticed but level of Scl-70 autoantibodies was not decreased. In 2011, the same team reported four supplementary cases of allogeneic BM-MSC systemic injection (0.22 to 1.8 × 10^6^ BM-MSCs/kg bodyweight) (79). Here again, improvement of vasculopathy and skin fibrosis was observed but the study underscored the need to set phase II clinical trials for efficacy assessment.

Local injection of autologous ASCs in combination with hyaluronic acid (HA) solution has been first tested in 2013 (63). Infiltration of 8 × 10^5^ ASCs/mL HA was done in a single area for each patient, either the face or the arm chosen with the patient consent. The procedure improved considerably skin fibrosis for four out of six patients and moderately for one patient. All patients showed arrest of local disease progression (100%), four of them presented regression of dyschromia (67%), five patients increased skin softening (83%), four patients showed better sensitivity (67%), and one patient reported erythema reduction (17%). The study demonstrated that ASCs could be successfully implanted locally in patients with severe disease, thus representing a good, feasible, and efficient cell-based soft-tissue augmentation strategy. Of interest, the same team thereafter compared autologous fat and ASC transplantation to evaluate clinical improvement of mouth opening in two groups of five patients (80). Both procedures obtained significant results but neither one emerged as a first-choice technique.

### Clinical Trials With MSCs

The results of the first open phase I clinical trial evaluating the interest of MSCs, and more precisely of MSC-containing SVF, on 12 SSc patients with severe functional hand handicap were published in 2015 (7). Injection of autologous SVF into fingers was safe and a significant improvement in pain, grasping capacity, finger edema, Raynaud's phenomenon, and quality of life was recorded at 6 months. At 12 months follow-up, a significant improvement in skin sclerosis, motion, edema and strength of the hand was observed. SVF was therefore claimed as a promising therapy whose effect persisted at least 1 year after injection (81). Finally, at 22 and 30 months after treatment, safety, tolerability and efficacy were very encouraging (82). The same team is now including 40 patients in a randomized double blind phase II trial to evaluate efficacy of the approach.

There are only 4 clinical trials recorded in the United State National library of Medicine (www.clinicaltrials.gov). One trial has been completed but no result is available. Only one of those is recruiting in France since 2014. This is a phase I/II trial which assesses the effect of one intravenous injection of intrafamilial allogenic MSCs on 20 patients. The trial is still enrolling patients and data are not yet available. Another phase I/II clinical trial has started in November 2017. It evaluates the therapeutic effect of allogeneic BM-MSCs after injection in intramuscular areas of affected limbs in 20 scleroderma patients with digital ulcers and is recruiting (NCT03211793) (83).

In summary, data from case reports and clinical trials are scare and need to be taken with cautious in terms of efficacy (Table 2). One question still pending is whether autologous or allogeneic ASCs or BM-MSCs have to be used in the clinics since some studies have reported phenotypic or functional alterations of MSCs from SSc patients.

**Table 2:** Summary of clinical trials using mesenchymal stem cells for the treatment of systemic sclerosis.

**Type of treatment**	**Route**	**Patient number**	**Clinical target**	**Clinical outcome**	**Adverse events**	**References**
Autologous fat grafting (mean of 30 ml/hand)	Subcutaneous in hand	9	Raynaud's phenomenon	Improvement of perfusion and decrease of ulcer numbers	None	(75)
Autologous fat grafting (0.5–1 ml/finger)	Subcutaneous in hand	15	Digital ulcers	Improvement of digital ulcerations, hand grasping and pain	None	(76)
Combined platelet-rich plasma and lipofilling treatment	Subcutaneous in peri-oral location	6	Face skin fibrosis	Improvement of skin elasticity, labial rhyme opening and vascularization	None	(77)
Allogeneic BM-MSCs (10^6^/kg bodyweight)	Intravenous	1	Systemic sclerosis	Reduction of ulceration and pain, improvement of hand vasculopathy	None	(78)
Allogeneic BM-MSCs (0.22–1.8 × 10^6^/kg)	Intravenous	5	Systemic sclerosis	Improvement of skin fibrosis and vasculopathy	Minor respiratory tract infection	(79)
Autologous ASCs (4 to 8 × 10^6^)	Subcutaneous peri-oral location	6	Localized skin scleroderma	Disease stabilization for all patients and improvement of skin elasticity in 4/6 patients	None	(63)
Autologous fat (16 ml) or ASCs (3.2 × 10^6^)	Subcutaneous peri-oral location	5	Face skin fibrosis	Improvement of skin fibrosis & mouth opening	None	(80)
Autologous SVF (5 ml/hand)	Subcutaneous in hand	12 (phase I trial)	Severe hand functional handicap	Improvement of pain, grasping capacity, finger edema, Raynaud's phenomenon, quality of life	None	(7) (81) (82)

## Conclusion

Many studies have now established the beneficial effect of the administration of BM-MSCs, ASCs or MSCs from other tissue sources in different preclinical models characterized by local or systemic fibrosis. Some evidence of safety and efficacy of MSC-containing SVF or culture expanded MSCs has been described from the clinics but efficacy needs to be further proved in phase II clinical trials that are ongoing. Both autologous and allogeneic MSCs from BM or adipose tissue are being assessed but the risk that the functional properties of MSCs isolated from SSc patients are altered is under debate. Contradictory results are reported in the literature but a number of reports discuss the reduction of the number of clonogenic cells, proliferative rate, differentiation, and angiogenic potentials. MSCs from SSc patients display a more mature and myofibroblast-like phenotype, probably related to microenvironmental signals dysregulated during the disease. They express higher levels of TβRII and TGFβ, which is released into the extracellular medium where it can act in an autocrine or paracrine manner. Moreover, the crosstalk between MSCs and ECs contribute to the altered expression of different molecules involved in angiogenesis, inducing a switch of perivascular MSCs toward a myofibroblast population, further supporting the fibrotic process. The finding that MSCs from SSc patients constitutively overexpress mediators involved in the fibrotic and angiogenic processes might indicate that MSCs are altered by the environment secondary to the onset of the disease or, that they might participate to the physiopathology of the disease. With respect to the use of autologous MSCs for clinical applications, further investigation on their functional properties is likely needed.

## Author Contributions

All authors listed have made a substantial, direct and intellectual contribution to the work, and approved it for publication.

### Conflict of Interest Statement

The authors declare that the research was conducted in the absence of any commercial or financial relationships that could be construed as a potential conflict of interest.
